# Regulating Circularly Polarized Light Detection via Polar‐Phase Transition in Alternating Chiral‐Achiral Cations Intercalation‐Type Hybrid Perovskites

**DOI:** 10.1002/advs.202307593

**Published:** 2023-12-27

**Authors:** Zeng‐Kui Zhu, Tingting Zhu, Shihai You, Panpan Yu, Jianbo Wu, Ying Zeng, Yuhang Jiang, Xitao Liu, Lina Li, Chengmin Ji, Junhua Luo

**Affiliations:** ^1^ State Key Laboratory of Structure Chemistry Fujian Institute of Research on the Structure of Matter Chinese Academy of Sciences Fuzhou 350002 China; ^2^ School of Chemistry and Chemical Engineering; Key Laboratory of Fluorine and Silicon for Energy Materials and Chemistry of Ministry of Education Jiangxi Normal University Nanchang 330022 China; ^3^ University of Chinese Academy of Sciences Beijing 100049 China; ^4^ Fujian Science and Technology Innovation Laboratory for Optoelectronic Information of China Fuzhou 350108 China

**Keywords:** alternating chiral‐achiral cations intercalation‐type hybrid perovskites, circularly polarized light detection, polar phase transition, polar photovoltaic effect, regulate anisotropy factor

## Abstract

Circularly polarized light (CPL) detection has wide applications in many fields, where the anisotropy factor (*g*_Iph_) is an important indicator to characterize the CPL detection performance. So far, many materials with high *g*_Iph_ have been reported, however, the exploration of the regulation of *g*_Iph_ is still in its infancy. Herein, two novel alternating chiral‐achiral cations intercalation‐type chiral hybrid perovskites (CHPs), named (*R*/*S*‐1‐phenylpropylamine)(propylamine)PbBr_4_ (1‐*R*/*S*), exhibit above room‐temperature (RT) polar‐phase transition, which greatly regulates the *g*_Iph_ value. The *g*_Iph_ of 1‐*R* is 0.04 in high‐temperature phase chiral non‐polar (*P*2_1_2_1_2_1_) by applying 5 V bias, interestingly, with the temperature decrease, the *g*_Iph_ value in low‐temperature phase chiral polar (*P*2_1_) gradually increases (0.22@360K, 0.40@340K, 0.47@320K), and finally reaches a maximum of 0.5 at RT. Such value is not only the highest among 2D CHPs to date, but presents a 12.5‐fold amplification compared with 0.04. Further, this rare phenomenon should be attributed to the built‐in electric field induced by the polar photovoltaic effect, which sheds light on further obtaining CHPs with large *g*_Iph_.

## Graphical abstract

Two novel alternating cations intercalation (ACI)‐type chiral hybrid perovskites with above room‐temperature (RT) polar phase transition are obtained. The *g*_Iph_ of 1‐*R* in high‐temperature phase chiral non‐polar structure is 0.04, and with the regulation of polar phase transition, the *g*_Iph_ gradually increases in low‐temperature phase chiral polar structure with the decrease of temperature and finally reaches 0.5 at RT.

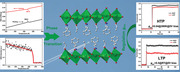

## 1 Introduction

Circularly polarized light (CPL) detection has received widespread attention because of its wide applications in quantum optics, drug screening, magnetic recording, and security monitoring.^[^ ^1^ ^]^ Recently, direct CPL detectors based on chiral materials have been favored for their distinguishability and direct detection of right‐hand CPL (RCP) and left‐hand CPL (LCP) without external optical elements, meeting the requirements for device miniaturization and integration.^[^ ^1^, ^2^ ^]^ The anisotropy factor of photocurrent (*g*_Iph_) is generally used to assess their distinguishability in CPL photodetection devices, which is closely related to their outstanding semiconductor properties and strong chiral optical activity.^[^ ^3^ ^]^ Therefore, it is critical to pursue direct CPL detectors with high *g*_Iph_.

Recently, chiral hybrid perovskites (CHPs), especially 2D CHPs, have been proven to be a very promising class of direct CPL detection materials,^[^ ^3^, ^4^ ^]^ such as (*R*)‐*β*‐MPA_2_MAPb_2_I_7_ (*g*_Iph_ of 0.11),^[^ ^4a^ ^]^ (*R*‐BPEA)_2_PbI_4_ (*g*_Iph_ of 0.13),^[^ ^4b^ ^]^ (*R*)‐*β*‐MPA_4_AgBiI_8_ (*g*_Iph_ of 0.3),^[^ ^4c^ ^]^ (*R*‐PPA)EAPbBr_4_ (*g*_Iph_ of 0.42),^[^ ^3b^ ^]^ and (*R*‐MBA)_2_Pb_0.9_Sn_0.1_I_4_ (*g*_Iph_ of 0.44).^[^ ^4f^ ^]^ Although there have been many reports of CHPs with large *g*_Iph_, however, how to effectively regulate and amplify *g*_Iph_ values also needs to be vigorously developed.

Changing polarity in CHPs may be an effective strategy for regulating the *g*_Iph_ values. Structural phase transition is one of the effective ways to regulate polarity.^[^ ^5^ ^]^ There have been many reported examples of changing polarity through phase transition regulation in non‐chiral hybrid perovskites.^[^ ^6^ ^]^ Considering that chirality is essential for achieving CPL detection, regulating polarity in CHPs is necessary. However, there are few studies of polarity regulation in CHPs, which are mainly in the regulation of ferroelectric properties.^[^ ^7^ ^]^ For example, Xiong reported the CHP [*R*/*S*‐1‐(4‐chlorophenyl)ethylammonium]_2_PbI_4_, which has a 422*F*1‐type ferroelectric phase transition from chiral non‐polar *P*422 to chiral polar *P*1 space group.^[^ ^7c^ ^]^ However, phase transitions modulating polar properties in chiral perovskites and the regulation of CPL detection *g*_Iph_ value have not been explored.

Herein, novel chiral alternating cations intercalation (ACI)‐type CHPs, named [(*R*/*S*)‐PPA]PAPbBr_4_ (1‐*R*/*S*, (*R*/*S*)‐PPA = *R*/*S*‐1‐phenylpropylamine; PA = propylamine), were obtained by using a cation alloying strategy. The differential scanning calorimetry (DSC), temperature‐dependent dielectric constant (εʹ), and second harmonic generation (SHG), and in situ variable‐temperature powder X‐ray diffraction (PXRD) jointly demonstrate that 1‐*R* has a reversible phase transition. Structure analysis shows that 1‐*R* achieves a chiral‐nonpolar (*P*2_1_2_1_2_1_) to chiral‐polar (*P*2_1_) change from the high‐temperature phase (HTP) to the low‐temperature phase (LTP), thereby the generated chiral polar space group brings a obvious polar photovoltaic effect (PPE) and a 0.23 V photovoltage at room temperature (RT). The 1‐*R* single‐crystal (SC) device in HTP can only operate with an external bias for photodetection, and a small *g*_Iph_ of 0.04 is obtained at a 5 V bias. Intriguingly, under the regulation of polar phase transition, the *g*_Iph_ of 1‐*R* gradually increases with the decrease of temperature (0.22@360K, 0.40@340K, 0.47@320K), and finally reaches 0.5 at self‐powered mode in RT, which is the highest *g*_Iph_ value reported in 2D CHPs to date. Thus, under the regulation of phase transition, 1‐*R* achieved a rare phenomenon of regulation and amplifying *g*_Iph_ (12.5‐fold amplification) for the first time, which shed light on further obtaining CHPs with large *g*_Iph._

## 2 Results and Discussion

### 2.1 Characterization Techniques and Crystal Structures

**Figure 1:**
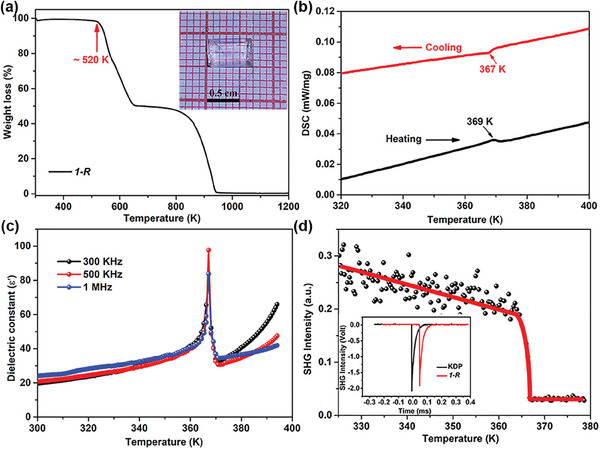
Phase transition properties of 1*‐R*. a) TGA curve, Inset: bulk crystal of 1‐*R*; b) DSC trace in heating and cooling processes; c) Temperature dependence of the dielectric constant; d) Temperature‐dependent variation of SHG optical signals. Inset: Comparison of SHG signals of 1‐*R* and KDP at RT.

Microcrystals of 1‐*R/S* were prepared by mixing Pb(OAc)_2_, *R*/*S*‐1‐phenylpropylamine (*R*/*S*‐PPA), and propylamine (PA) in a hot concentrated HBr solution. After a slow cooling process, bulk crystals (1‐*R*, 7 × 4 × 3 mm^3^) were obtained (see inset in **Figure** **1**a). The PXRDs show that 1*‐R/S* matches well with the simulated diffraction peaks, indicating its phase purity (Figure S1, Supporting Information). Furthermore, 1 exhibits a high thermal stability up to 520 K, which is evidenced by the thermogravimetric analysis (TGA) curve (Figure 1a). DSC measurements show the presence of two thermal peaks at 367 and 369 K (Curie temperature, *T*_c_) during the cooling and heating processes, respectively (Figure 1b), indicating a reversible phase transition of 1‐*R*.^[^ ^6c^ ^]^ Moreover, the phase transition of 1*‐R* is also confirmed by the significant anomaly around *T_c_* in its εʹ measurement (Figure 1c). For convenience, we refer to the phase below *T*_c_ as the LTP and the phase above *T*_c_ as the HTP. Besides, a distinct SHG intensity peak in RT is 0.92 times that of KDP (see inset in Figure 1d), emphasizing that 1 belongs to a non‐centrosymmetric space group in LTP. As the temperature increases, the SHG signal drops significantly above *T_c_* (Figure 1d), which can confirm the occurrence of a phase transition with symmetry breaking.^[^ ^6a,b^ ^]^ Furthermore, in situ variable‐temperature PXRD measurements also demonstrate a significant reversible phase transition of 1‐*R* near *T*_c_ (detail see Figure S2, Supporting Information).

**Figure 2:**
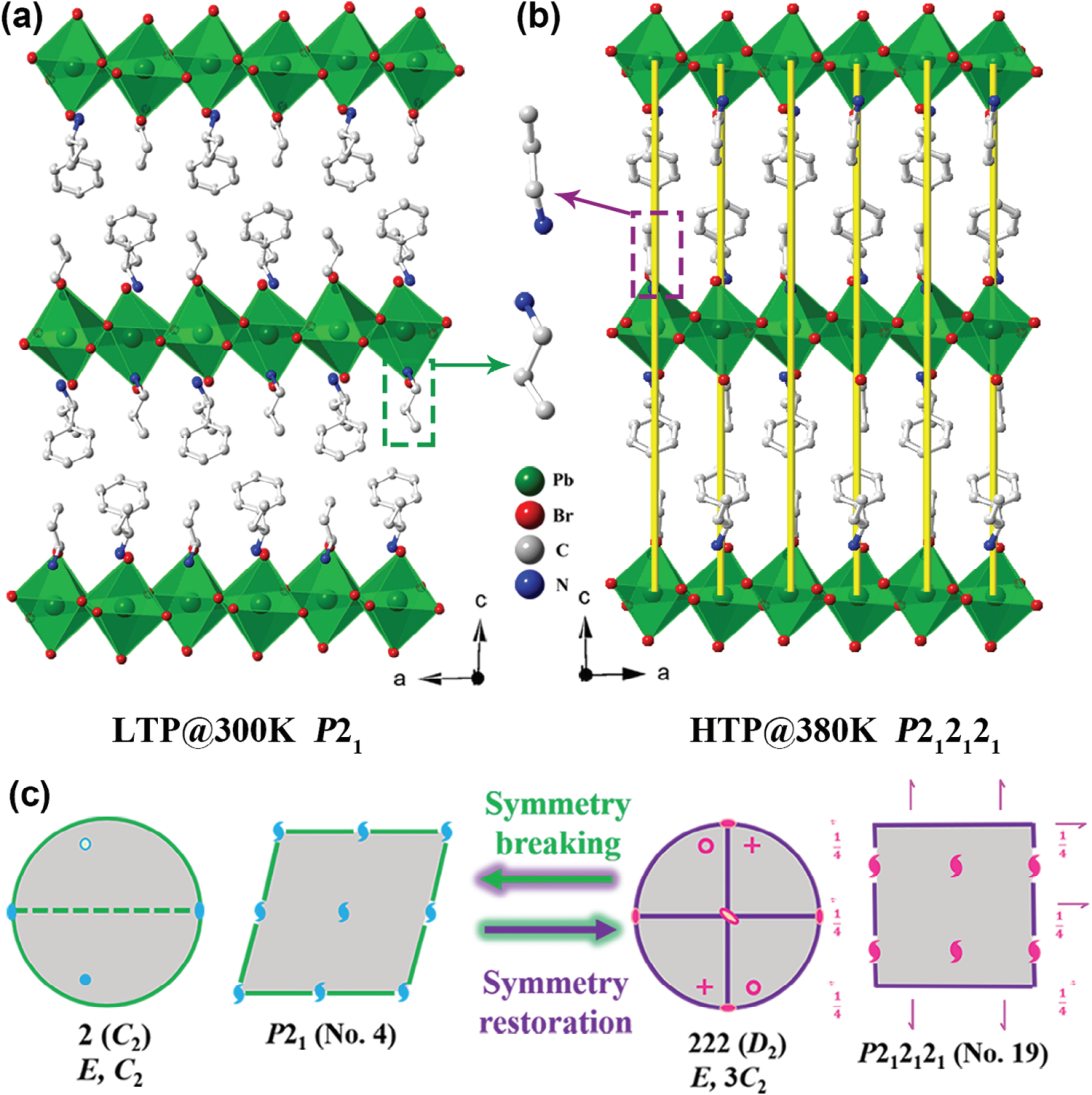
a,b) Packing structure of 1‐*R* in LTP and HTP, respectively, the yellow lines present the 2_1_ helix axes along the *c*‐axis in HTP; c) Equatorial plane projection of point groups and spatial symmetry operations of space groups in LTP and HTP. PbBr_6_ octahedra: Medium green.

To further clarify the phase transition mechanism, the structures of 1‐*R*/*S* in different phases are determined by single‐crystal X‐ray diffraction. Since 1*‐R* and 1*‐S* are antisymmetrical isostructural (Figure S3, Supporting Information), 1‐*R* is chosen for depicting the LTP and HTP crystal structure in detail. Structure analysis reveals that 1‐*R* is a 2D monolayered ACI‐type CHP, which crystallizes in a monoclinic system with the chiral polar space group of *P*2_1_ in LTP. 1*‐R* is composed of the inorganic layers connected by corner‐sharing octahedral PbBr_6_ and two organic cations (including chiral aromatic PPA^+^ and achiral chain PA^+^ cation) arranged between interlayer space (**Figure** **2**a). Specifically, four adjacent Pb^2+^ ions are bridged by Br^−^ ions to form a distorted square on the *ab* plane, where the bond length of Pb‐Br is 2.920‐3.103 Å and the bond angle of Pb‐Br‐Pb is 142.074–155.95°, respectively (Figure S4, Supporting Information). Besides the inorganic skeletons, both PPA^+^ and PA^+^ cations are alternately arranged in the interlayer space and opposite in a 1:1 manner along the *c*‐axis (Figure 2a). Further, these cations are firmly anchored between two inorganic perovskite skeletons through N‐H⋅⋅⋅Br bonds (Figure S5, Supporting Information), resulting in the formation of 1‐*R* (Figure 2a).

As the temperature increases above *T*_c_, 1‐*R* crystallizes in the orthorhombic system of the chiral non‐polar space group *P*2_1_2_1_2_1_ (Figure 2b). Although no obvious changes are found in the inorganic skeleton, the thermal ellipsoids of organic cations tended to increase relative to LTP, suggesting the possibility of an ordered‐disordered transition (Figure S6, Supporting Information). To further compare the changes of the polar axis in LTP and HTP, the 2_1_ helical axes of LTP and HTP are displayed in detail (Figure 2b and Figure S7, Supporting Information). It can be seen that the short‐chain PA^+^ in HTP (Figure S8a,c, Supporting Information) is closer to a straight chain than the twisted state under LTP (Figure S7a,c, Supporting Information), which may account for its loss of the 2_1_ helical axis along *a*‐ and *c*‐ axis in LTP (Figure 2a,b and Figure S7, Supporting Information). The PA and PA cations are arranged approximately in parallel, which also satisfies the symmetry requirement of non‐polar space groups through cation reorientation^[^ ^6c^ ^]^ (Figure 2a,b). Thus, a symmetry breaking with 222*F*2 as the Aizu symbol is found, which fits one of the 88 types of ferroelectric symmetry‐breaking^[^ ^6a,b^ ^]^ (Figure 2c). Therefore, the DSC, variable‐temperature dielectric and SHG, and structure analysis indicate that 1*‐R* is a potential ferroelectric material, and the crystal symmetry achieves the transition from HTP chiral non‐polarity to LTP chiral polarity.

### 2.2 Optical Properties

**Figure 3:**
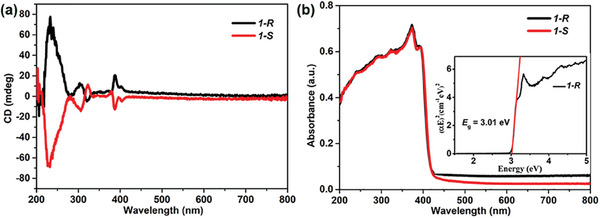
a) Circular dichroism spectrum of 1*‐R/S* films; b) Solid‐state diffuse reflectance UV–vis spectrum of 1*‐R/S*. The optical bandgap of 1*‐R* revealed in the inner window.

Considering the crystallization in the chiral space group, the chiral performance of 1 was studied further. As shown in **Figure** **3**a, the 1‐*R* and 1‐*S* films show a distinct circular dichroic (CD) signal, which is observed at the same wavelength (at 233, 304, 323, 387, and 404 nm) but with opposite values.^[^ ^3^, ^8^ ^]^ Compared to the CD signals of the chiral ligands *R*/*S*‐PPA (at 280 nm in the range of 200–300 nm),^[^ ^3^, ^4^ ^]^ the CD signals of 1‐*R*/*S* were significantly expanded to longer wavelengths (300–404 nm), which also demonstrates the successful transfer of chirality from the chiral ligands to the perovskite frameworks.^[^ ^3^, ^4^, ^8^ ^]^ The PXRDs of the thin films show that the films have a high degree of orientation along the (*0 0 l*) direction (Figure S9, Supporting Information). The corresponding calculated CD anisotropy factor (*g*_CD_ = CD/(32980 × absorbance)) is about 0.0048 at 233 nm for 1‐*R* (Figure S10, Supporting Information), which is comparable to some known chiral perovskites.^[^ ^3^, ^4^ ^]^ Furthermore, the value of g_CD_ is about 7.44 × 10^−4^ at 405 nm, which is comparable to [4APEA]PbI_4_ (g_CD_ = 1 × 10^−4^).^[^ ^4g^ ^]^ These results demonstrate the inherent chiral optical activity of 1. The difference in PL intensity between 1‐*R* and 1‐*S* crystals at 77 K has been measured under 405 nm left‐handed (σ^−^) and right‐handed (σ^+^) CPL excitation with a power of 125 µW (Figure S10a,b, Supporting Information). The obvious intensity difference can be observed between the σ^−^ and σ^+^ CPL light in 490 nm for the chiral 1‐*R* and 1‐*S* crystals (Figure S10a,b, Supporting Information). To further quantify the degree of the circularly polarized PL, a parameter *P* was introduced, which is defined as *P* = (*I*_left_ − *I*_right_)/(*I*_left_ + *I*_right_), wherein *I*_left_ and *I*_right_ represent the intensity of the left‐ and right‐handed circularly polarized PL, respectively. The estimated /P/ of 1‐*R* and 1‐*S* crystals are 16.6% and 18.3%, respectively. These values are comparable to that of pure chiral 2D perovskites R/S‐α‐(PEA)_2_PbI_4_ measured at 77 K (10%) and room temperature (13.7%)^[^ ^8c,d^ ^]^ and much larger than that in the chiral reduced‐dimensional perovskites at 2 K (3%).^[^ ^8e^ ^]^ Moreover, solid‐state diffuse reflectance UV–vis spectrum (DRS) exhibits that the extinction band edge of 1*‐R* is around 420 nm, and the optical band gap is determined to be 3.05 eV. Further, the theoretically calculated electron band structure shows that 1‐*R* features a direct bandgap (Figure S11a, Supporting Information). The calculated bandgap of 1‐*R* is 2.76 eV, which is comparable to the experimental value. The partial density of states shows that the electronic structure and optical bandgap of 1‐*R* are mainly dominated by the inorganic skeleton derived from the Pb‐6p and Br‐4p orbitals, which is further supported by the charge density distribution of VBM and CBM^[^ ^9^ ^]^ (Figure S11b,c, Supporting Information). These characteristics, such as chiral, polar, and direct bandgap semiconductors, suggest that 1‐*R* is suitable for CPL detection.^[^ ^10^ ^]^

### 2.3 CPL Detection and Regulation of *g*_Iph_

**Figure 4:**
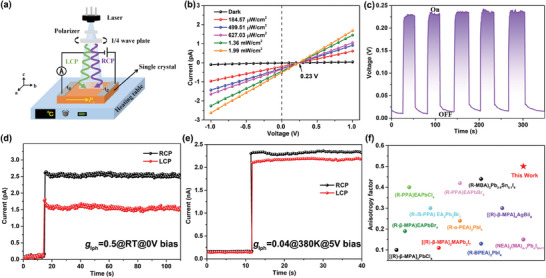
a) The schematic of single crystal device; b) *I*–*V* curve in the dark and light illumination with varied intensities at RT; c) *V‐*‐*t* curve at RT; d,e) CPL measurement at 0 V‐ and 5 V‐biases under 288 mW cm^−2^ in LTP and HTP, respectively; f) The anisotropy factors of some reported 2D chiral hybrid perovskites.

The polar axis of 1‐*R* in LTP is on the *b*‐axis, which is verified by the electron polarization value of 2.291 nC cm^−2^ calculated by the point charge model along the *b*‐axis^[^ ^11^ ^]^ (Figure S12, Supporting Information). So the Ag/ 1‐*R* SC /Ag electrode devices formed along the *b*‐axis were used to probe the possible influence of polarity on their optoelectronic properties (**Figure** **4**a). Firstly, the photoconductivity of 1‐*R* in the HTP phase was investigated, and the device temperature was raised to 380 K (> *T*_c_) by a variable temperature heating stage. Figure S13, Supporting Information exhibits the current–voltage (*I*–*V*) curves of the 1‐*R* device in the dark and under 405 nm light illumination. 1‐*R* presents a relatively high dark current of 0.38 nA at 10 V bias. As the optical power density increases, the photocurrent increases to 4.68 nA at 682.16 mW cm^−2^ with a switching ratio of 12. The inability to measure the current–time (*I‐*‐*t*) curve at 0 V bias indicates that 1‐*R* cannot achieve self‐powered mode in HTP. Therefore, 5 V bias is added, and the results exhibit that the current increases as the optical power density increases (Figure S14, Supporting Information). Further, the CPL detection of 1‐*R* was tested under 5 V bias. As shown in Figure 4e, the photocurrent of RCP is larger than that of LCP under the same light intensity (288 mW cm^−2^), revealing the ability to distinguish between RCP and LCP. Accordingly, the ability to distinguish CPL is evaluated by the *g*_Iph_, which is derived from the formula *g*_Iph_ = 2(*I*_R_− *I*_L_)/(*I*_R_ + *I*_L_), where *I*_R_ and *I*_L_ represent the photocurrent under 405 nm RCP and LCP irradiation. The *g*_Iph_ value is calculated to be a relatively small 0.04 at 5 V bias (Figure 4e). For comparison, the 1‐*R* device was subjected to CPL detection at room temperature at 5 V bias with a g_Iph_ of 0.1 (Figure S19c, Supporting Information). These values are smaller than that of many reported CHPs^[^ ^3^, ^4^ ^]^ (Table S4, Supporting Information).

To further investigate the effect of phase transition on material photoconductivity and CPL detection, the same 1‐*R* device is progressively cooled to RT. Interestingly, as the temperature drops below *T*_c_, the voltage value in the voltage–temperature (*V–T*) curve starts to increase gradually and eventually reaches a maximum of 0.23 V at RT, (Figure S15, Supporting Information) which should be attributed to the PPE.^[^ ^3^, ^4^ ^]^ Typically, the PPE is closely associated with the built‐in electric fields in CHPs that can spontaneously separate photogenerated electron and hole pairs, giving the material great potential for self‐powered CPL detection.^[^ ^12^ ^]^ Moreover, the 1‐*R* device further tested its photoconductivity and CPL detection at RT. The *I*–*V* curves of 1‐*R* are shown in Figure S16, Supporting Information. Interestingly, 1‐*R* exhibits an extremely low dark current (≈3.2 pA) at 10 V bias, which is 118 times smaller than that of HTP. Such a low dark current indicates that 1‐*R* SC has a high quality and a low intrinsic carrier concentration, which facilitates high‐performance photodetection. Further, the photocurrent increases sharply under 405 nm light irradiation and eventually rises to 3.68 nA at a light intensity of 576.08 mW cm^−2^ (Figure S16, Supporting Information). This considerable photocurrent and ultra‐low dark current thus generate a large on/off ratio of 1.1 × 10^3^, a value about 100 times larger than that in HTP. Noteworthy, 1‐*R* has significant photocurrent generation at a very weak photocurrent density of 99.5 nW cm^−2^ (see inset in Figure S16, Supporting Information), which is comparable to those of some excellent 2D perovskite materials, for example, (BA)_2_PbBr_4_ (80 nW cm^−2^) and (FPEA)_2_(MA)Pb_2_I_7_ (100 nW cm^−2^).^[^ ^13^ ^]^ Moreover, the 0.23 V photovoltage at RT has also been verified by *I–V* and the time‐dependent voltage (*V‐*‐*t*) curves (Figure 4b,c), and the photovoltaic can remain stable after multiple on/off cycles (Figure 4c), which prompted us to investigate its self‐powered photoelectric detection.

Figure S17, Supporting Information shows the *I*–*t* curve of 1‐*R* at different light intensities under 0 V bias. The results present that the current increases gradually with the increase of light intensity, indicating that the material has excellent photoresponse stability under self‐powered mode (0 V bias). Furthermore, the self‐powered CPL detection performance of 1‐*R* has been studied. As shown in Figure 4d and Figure S18, there is a significant increase in *g*_Iph_ value as the temperature decreases below *T*_c_ (0.22@360K, 0.40@340K, 0.47@320K), and finally reaches a maximum *g*_Iph_ value of 0.5 at RT. The g_Iph_ value is much larger than that of g_CD_, a phenomenon that has been reported in many chiral perovskite materials, which could be attributed to spin‐dependent carrier transport and collection.^[^ ^3^, ^4^ ^]^ This phenomenon indicates that the chiral polarity change of 1‐*R* in the structure is regulated by the polar phase transition process, and thus the magnitude of the *g*_Iph_ value is successfully adjusted. In addition, compared to a *g*_Iph_ value of 0.04 for HTP, this value has a 12.5‐fold amplification to 0.5 for the RT phase, which is also the highest value among 2D CHPs reported so far^[^ ^3^, ^4^ ^]^ (Figure 4f, and Table S4, Supporting Information). Such large value may be caused by the aspect that the PPE in chiral perovskites is CPL‐dependent, and the photovoltaic response in different CPL states differs significantly when irradiated with left‐ and right‐handed light.^[^ ^4d^ ^]^ Therefore, the driving force provided by the PPE is distinct under the excitation of different helicities. By combining the intrinsic chirality, chiral‐induced spin selectivity, and CPL‐dependent PPE, our device finally achieves a highly sensitive CPL detection with a large anisotropic factor of 0.5.^[^ ^4d^ ^]^ This interesting phenomenon shows that under the influence of phase transition (HTP to LTP), not only does the reversible change of the material from chiral‐non‐polar to chiral polarity be achieved, but regulate and amplify the *g*_Iph_ value from 0.04 to 0.5 (12.5‐fold amplification), which is also an important reference for constructing CHPs with high *g*_Iph_. Furthermore, the stability of the 1‐*R* device in a working state can be proved by cycle‐repeating experiments at RT and long‐term photocurrent stability at 380K @5 V bias (Figure S20, Supporting Information).

## 3 Conclusion

In summary, two novel chiral ACI‐type perovskite materials 1‐*R*/*S* were obtained. Many characterization techniques and crystal structure analyses show that 1‐*R* undergoes a chiral non‐polar (*P*2_1_2_1_2_1_) to chiral polarity (*P*2_1_) structural transition process from HTP to LTP, and the generated chiral polar space group brings the PPE and 0.23 V photovoltage in RT phase. Further, the optoelectronic performance measurements show that 1‐*R* can only operate at a certain applied voltage at HTP, and a small *g*_Iph_ of 0.04 is obtained under a 5 V bias. Surprisingly, with the effect of PPE and photovoltage at self‐powered mode, the *g*_Iph_ value of 1‐*R* gradually increases with the decrease of temperature, and finally reaches 0.5 at RT, which is not only by far the maximum value among 2D CHPs, but also presents a 12.5‐fold amplification compared with that of 0.04 in HTP. Thus, a rare *g*_Iph_ regulation and amplification phenomenon (12.5‐fold amplification) was achieved for the first time by 1‐*R* under the regulation of polar phase transition, which also provides a reference for further obtaining CHPs with large *g*_Iph_.

## Conflict of Interest

The authors declare no conflict of interest.

## Supporting information

Supporting Information

## Data Availability

The data that support the findings of this study are available from the corresponding author upon reasonable request.
