# Supplementary material for: Regulating Circularly Polarized Light Detection via Polar‐Phase Transition in Alternating Chiral‐Achiral Cations Intercalation‐Type Hybrid Perovskites
Source: Adv Sci (Weinh). 2023 Dec 27;11(6):2307593. doi: 10.1002/advs.202307593 (PMC10853736; doi:10.1002/advs.202307593)
Supplement: Supplementary file 1 — Supporting Information [file ADVS-11-2307593-s001.pdf]

ADVANCED SCIENCE
Open Access

Supporting Information

for *Adv. Sci.*, DOI 10.1002/advs.202307593

Regulating Circularly Polarized Light Detection via Polar-Phase Transition in Alternating Chiral-Achiral Cations Intercalation-Type Hybrid Perovskites

*Zeng-Kui Zhu, Tingting Zhu, Shihai You, Panpan Yu, Jianbo Wu, Ying Zeng, Yuhang Jiang, Xitao Liu, Lina Li, Chengmin Ji and Junhua Luo**

Supporting Information

Regulating Circularly Polarized Light Detection *via* Polar-Phase Transition in Alternating Chiral-Achiral Cations Intercalation-type Hybrid Perovskites

*Zeng-Kui Zhu, Tingting Zhu, Shihai You, Panpan Yu, Jianbo Wu, Ying Zeng, Yuhang Jiang, Xitao Liu, Lina Li, Chengmin Ji, Junhua Luo**

Experimental Section and Discussions

Materials. Lead(II) acetate trihydrate ($Pb(OAc)_2·3H_2O$, 99.5%, AR), Hydrobromic acid (HBr, 48%), (*R*)-(+)-1-Phenylpropylamine reagent (*R*-PPA), (*S*)-(-)-1-Phenylpropylamine reagents (*S*-PPA), n - propylamine (PA, 99%, GC) are used directly from Aladdin without further purification.

Synthesis of (*R*-PPA)PAPbBr$_4$ (*1-R*): single crystals were synthesized by continuously heating and stirring the mixing $Pb(OAc)_2·3H_2O$ (2 mmol), *R*-1- phenylpropylamine (2 mmol), *n* - propylamine (2 mmol) and aqueous HBr (48%, 10mL) in a glass vial. Single crystals were grown through the temperature lowering method from a saturated solution in the range of 40°C to 45°C. Before the growth commencement, the saturated solution was kept at 80 °C for 3 hours. With the solution temperature decreasing to saturated status, crystals were obtained by spontaneous nucleation. The growth of large-sized crystals is mainly obtained by slow cooling mothed in an oven, and the cooling rate is set at 0.5 K/day. High-quality red crystals could be gained after several days.

Synthesis of (*S*-PPA)PAPbBr$_4$ (*1-S*): single crystals were synthesized by continuously heating and stirring the mixing $Pb(OAc)_2·3H_2O$ (2 mmol), *S*-1- phenylpropylamine (2 mmol), *n* - propylamine (2 mmol) and aqueous HBr (48%, 10 mL) in a glass vial. Single crystals were grown through the temperature lowering method from a saturated solution in the range of 40°C to 45°C. Before the growth commencement, the saturated solution was kept at 80 °C for 3 hours. With the solution temperature decreasing to saturated status, crystals were obtained by spontaneous nucleation. The growth of large-sized crystals is mainly obtained by slow cooling mothed in an oven, and the cooling rate is set at 0.5 K/day. High-quality red crystals could be gained after several days.

Instruments: PXRD were performed on the MiniFlex 600 X-ray diffractometer equipped with a Cu Kα radiation. The diffraction patterns were collected in the 2θ range of 5 ~ 50° with

a step size of 0.05°. The UV-vis absorptions in the solid-state are measured at room temperature on a PE Lambda 950 UVVisible spectrophotometer with $BaSO_4$ as blank. Thermogravimetric analysis (TGA) measurement was conducted on a Netzsch STA449C thermal analyser in the temperature range of 30~900 ^{o}C and recorded at heating rate of 10 ^{o}C min^{-1}. The differential scanning calorimetry (DSC) was performed on the NETZSCH DSC 200 F3 with the heating/cooling rates of 10 K/min under the N_2 atmosphere. The dielectric analyses were performed on TongHui TH2828 analyzer, and the single crystals of 1-*R* with the surface deposited by silver paste were used for dielectric constant measurements. Second harmonic generation (SHG) measurement are performed on powder samples using a Nd:YAG laser (λ=1064 nm, 5 ns pulse duration, 1.6 MW peak power, 10 Hz repetition rate). The *in situ* variable-temperature powder X-ray diffraction (PXRD) was performed by the Rigaku Ultima IV.

Single crystal structure determination: Crystal **1-*R*** at LTP was collected on a Bruker APEX Due CCD area diffractometer equipped with a fine focus, and with a 2.0 kW sealed tube X-ray source (MoKα radiation, λ = 0.71073 Å) operating at 300 K. Crystals 1-*R* at HTP was collected on a Rigaku XtaLAB Synergy-R MoKα radiation (λ = 0.71073 Å) at 380 K. And crystal **1-*S*** was collected on ROD, Synergy Custom system, HyPix diffractometer with micro-focus metaljet Kα (λ = 1.34050 Å) radiation at 100 K. The empirical absorption correction was based on equivalent reflections. Structures were solved by direct methods followed by successive difference Fourier methods. Computations were performed using SHELXTL and final full-matrix refinements were against F2. All the non-hydrogen atoms were anisotropically refined. The positions of hydrogen atoms of organic moieties were generated geometrically. CCDC 2253290-2253292 contain the supplementary crystallographic data for compounds **1-*R*** at LTP and HTP and **1-*S***, respectively.

Circular dichroism (CD) spectrum: A Bio-Logic MOS450 CD spectrometer is used for the CD measurements.

Film: The films of *1-R/S* are fabricated by spin coating method with quartz glass used as substrate. Substrates with desired dimension are cleaned in an ultrasonic cleaner using deionized water, isopropanol, and acetone in sequence for 20 minutes of each. Next, the substrate surface is cleaned by ultraviolet-ozone cleaner for 30 minutes. The precursor solutions for the chiral perovskites are prepared by dissolving the *1-R* and *1-S* microcrystals in anhydrous DMF (0.06 mol/L) for CD measurements. To form the films, 100 μL of the precursor solution is spread on the cleaned surface of the substrate, and then spun at 1000 rpm for 15 s, 2000 rpm for 30 s. Finally, the as-fabricated films are annealed at 75 ^{o}C for 15

minutes on a hot-plate to induce crystallization. The resulting film size is approximately $2 \times 2 \times (1\times10^{-4})$ cm^3.

Theoretical calculation: Single crystal data of *1-R* is used as an example for the theoretical caculations. Band structure and partial density of states (PDOS) are calculated using the DFT method within the total energy code CASTEP. The exchange and correlation effects are treated by the Perdew-Burke-Ernzerh method of generalized gradient approximation. The core-electrons interaction between the ion cores and the electrons are described in terms of the norm-conserving pseudopotential.

Photoelectric and circular polarized light (CPL) measurements: Photoelectric measurements were performed with a planar electrode configuration. The current *vs* voltage (*I-V*) with the light on or off were measured using a high precision electrometer (Keithley 6517B). A THORLABS 405 nm pigtailed laser diode (LP405-MF100, 105 mW) was used for visible light illumination. The incident light intensity was measured by light power meter. The temperature during measurements was controlled at 298 K using a Linkam TS1500 heating stage. CPL measurements are applied based on a linear polarizer and a quarter-wave plate on crystal ***1-R***. The pump is right-handed circularly polarized when the rotation angles are 45°and 225°, and is left-handed circularly polarized when the rotation angles are 135° and 315°, respectively.

Discussion for the advantages of 2D chiral hybrid perovskite: As we all know, chiral hybrid perovskite materials are significant and many chiral hybrid perovskites with high g_{Iph} factors have been reported. Specifically, 3D perovskites with the structural formula of ABX_3 are limited by the Goldschmidt tolerance factor of the A-site cation, so it is structurally difficult to obtain 3D chiral hybrid perovskites while the chiral cations are much bigger than the typical A-site cations such as methylamine, formamidine, and cesium the inherent structure instability and ionic migration of 3D hybrid perovskites further hinders their practical optoelectronic applicability. (*Adv. Mater.* **2021**, *33*, 2008785; *Chin. Phys. Lett.* **2018**, *35*, 036104) Compared with 3D perovskites, 2D perovskites do not have the restriction of the Goldschmidt tolerance factor, so varieties of chiral cations can be introduced, leading to the significant designability of their structures. So far, many chiral organic ligands have been reported for the synthesis of 2D and quasi-2D chiral perovskites. (*Adv. Mater.* **2021**, *33*, 2008785; *Adv. Funct. Mater.* **2023**, 2306199) In addition, by varying the number of layers “n” and chemical composition, the optical and electrical properties of 2D chiral perovskites can be easily modulated which is beneficial for optoelectronic applications. Besides the structural

diversity, 2D CHPs often exhibit large anisotropic absorption of circularly polarized light, which facilitates the design of chiral perovskites with large g_{Iph} factors. Furthermore, 2D CHPs are generally characterized by symmetry breaking of structure, spin polarization, Rashba-Dresselhaus spin-orbit coupling, etc., which are equally favorable for obtaining 2D CHPs with high g_{Iph}. (*Nat. Photonics* ***2018****, 12,* 528; *Adv. Funct. Mater.* 2023, 2306199; *Nat. Commun.* **2020**, *11*, 4699) Compared with 2D chiral perovskites, 1D and 0D counterparts generally exhibited poor semiconductor properties, owing to poor carrier transport. Therefore, the current works based on 1D and 0D perovskite devices for CPL detection are still relatively scarce, and still need further research and exploration. (*Sci. Adv.* 2020, **6**: eabd3274; *Chem. Mater*. **2022**, *34*, 2955-2962; *Giant*, **2022**, *9*, 100086; *Angew. Chem. Int. Ed.* **2021**, *60*, 20021-20026; *Nano Lett.* **2023**, *23*, 606-613; *Nano Lett.* **2022**, *22*, 846-852)

Discussion for the synthesis strategy of the novel chiral ACI-type perovskites: In order to overcome the reality that the suitable and relatively limited number of commercial chiral cations restricts the rapid development of chiral hybrid perovskites, we initially focused on how to design fruitful range of chiral hybrid perovskites by selecting a specific chiral cation and incorporating a wide variety of achiral cations through **a cation alloying strategy**, which ultimately obtained novel 2D chiral ACI-type hybrid perovskites. After extensive experimental exploration, we found that the *R*/*S*-PPA cation is a suitable cation for the construction of novel ACI chiral hybrid perovskites. And by using **a cation alloying strategy**, a series of 2D lead-based chiral ACI-type hybrid perovskites based on the chiral cation *R*/*S*-PPA were obtained, such as (*R*/*S*-PPA)(*A*)PbX_4 (*A* = EA, F-EA, Cl-EA, Br-EA, I-EA, PA, Cl-PA, Br-PA, I-PA, allylamine, propargylamine, butylamine, pentylamine, iso-pentylamine, *etc*.; X = Br, Cl); (*R*/*S*-PPA)$(EA)_2Pb_2Br_7$; (*R*/*S*-PPA)(A′)(MA)Pb_2Br_7 (A′ = EA, PA, butylamine, *etc*.). They crystallize in different chiral space groups and exhibit different and appealing optoelectronic performance, holding great promise for application in future chiroptoeletronics and spintronics. And some works have been reported. (*Mater. Horiz.*, **2023**, *10*, 5307-5312; *J. Am. Chem. Soc.* **2022**, 144, 18062-18068; *Small* **2022**, *18*, 2203571; *Adv. Optical Mater.* **2022**, 2200146)

Discussion for the effects of cation alloying on the crystal structure and optoelectronic properties: Firstly, for the impact on the crystal structure of chiral hybrid perovskites, the use of **a cation alloying strategy** is an effective strategy for constructing new chiral ACI-type perovskites. Secondly, the **cation alloying strategy** is an effective approach that induces the

crystallization of the material in polar space groups and gives rise to the bulk photovoltaic effect. For example, with only a single chiral cation, *R*/*S*-PPA or *R*/*S*-MPA, 1D chain (*R*/*S*-PPA)$PbBr_3$ and 2D monolayered RP-type (*R*/*S*-MPA)$_2PbBr_4$ chiral hybrid perovskite materials can be obtained, respectively, both of which are crystallized in the chiral non-polar space group $P2_12_12_1$, and therefore neither of them possesses a bulk photovoltaic effect. (*J. Am. Chem. Soc.* **2022**, *144*, 18062-18068; *J. Am. Chem. Soc.* **2021**, *143*, 43, 18114-18120; *Adv. Sci.* **2023**, 2206070) However, when introduce a second, low-symmetry, chain amine cation such as ethylamine (EA) and propylamine (PA), the compounds obtained, such as (*R*/*S*-PPA)$EAPbBr_4$, (*R*/*S*-PPA)$PAPbBr_4$, (*R*/*S*-MPA)$EAPbBr_4$, (*R*/*S*-MPA)$PAPbBr_4$, etc., were all crystallized in the lower-symmetry chiral polar space group $P2_1$, which also induced them have bulk photovoltaic effect and higher g_{Iph} values. (*J. Am. Chem. Soc.* **2022**, *144*, 18062-18068; *Adv. Sci.* **2023**, 2206070) Thirdly, the bandgap of obtained ACI-type perovskites becomes slightly smaller compared to RP-type chiral hybrid perovskites constructed with single chiral cation, (*Adv. Sci.* **2023**, 2206070) and further, the addition of halogen-substituted achiral cations as well as the construction of 2D multilayered chiral ACI-type hybrid perovskites can greatly expand the absorption range of the materials and further reduced its bandgaps. (*J. Am. Chem. Soc.* **2022**, *144*, 18062-18068; *Small* **2022**, *18*, 2203571) And chiral ACI perovskite materials will have significantly different locations of CD signals compared to single chiral RP-type or 1D chain-like chiral hybrid perovskites. (*J. Am. Chem. Soc.* **2022**, *144*, 18062-18068; *Small* **2022**, *18*, 2203571; *Mater. Horiz.*, **2023**, *10*, 5307-5312) From the results of the reported data and the analysis of our data, we found that the g_{CD} values are mostly in the same order of magnitude. (*J. Am. Chem. Soc.* **2022**, *144*, 18062-18068; *J. Am. Chem. Soc.* **2021**, *143*, 43, 18114-18120; *Small* **2022**, *18*, 2203571) Last but not least, ACI-type perovskite has a flatter skeleton, which leads to better carrier transport properties and further endows the materials with excellent photodetection properties, such as good photoresponsivity and high g_{Iph}.

Figures

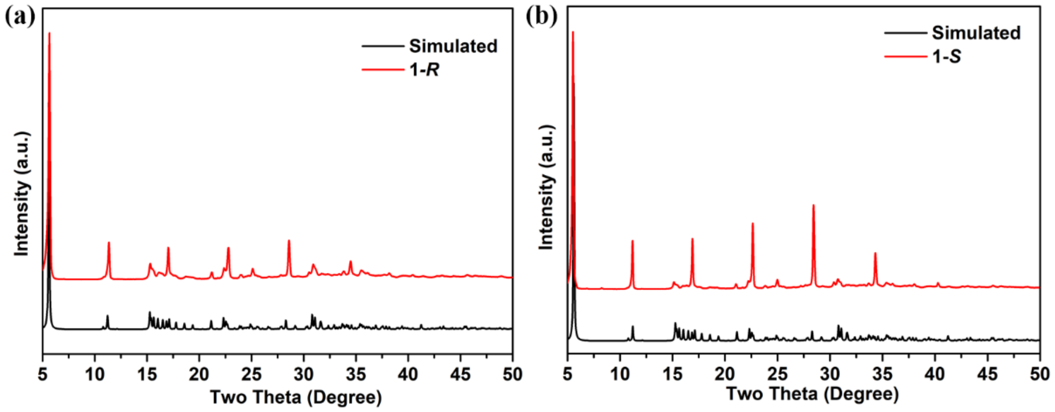

Figure S1. Powder X-ray diffraction patterns of (a) **1-*R***; (b) **1-*S***.

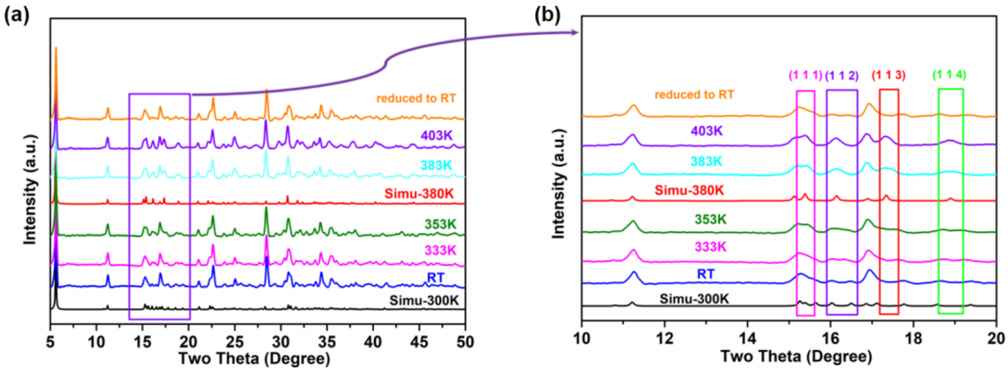

Figure S2. *In situ* variable-temperature powder X-ray diffraction of (a) ***1-R*** at the range of 5-50°; (b) ***1-R*** at 10-20°.

In situ variable-temperature PXRD confirms that the diffraction peaks on the (1 1 1), (1 1 2), (1 1 3), and (1 1 4) crystal plane of HTP gradually appear and intensify when the temperature reaches above T_c, and these new peaks also indicate that the crystals have undergone a phase transition. (Figure S2) When the temperature is cooled down to room temperature (RT) again, PXRD reverts to match the LTP phase, which indicates that **1-*R*** has a reversible phase transition.

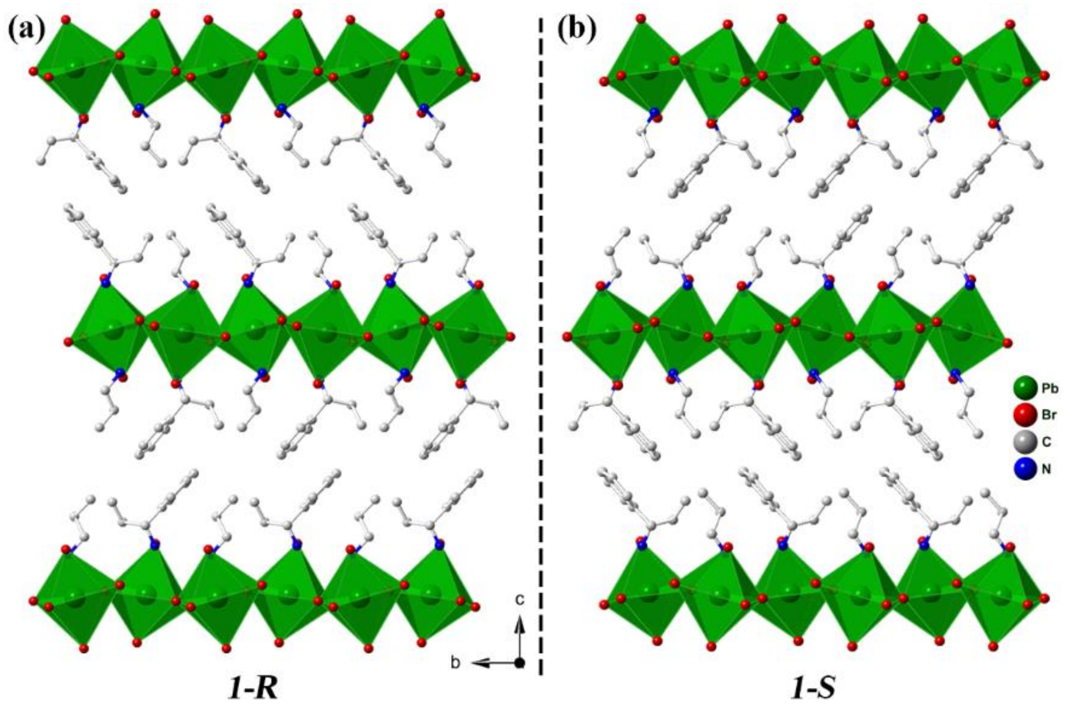

Figure S3. Structure of **1-*R*** and **1-*S*** in LTP.

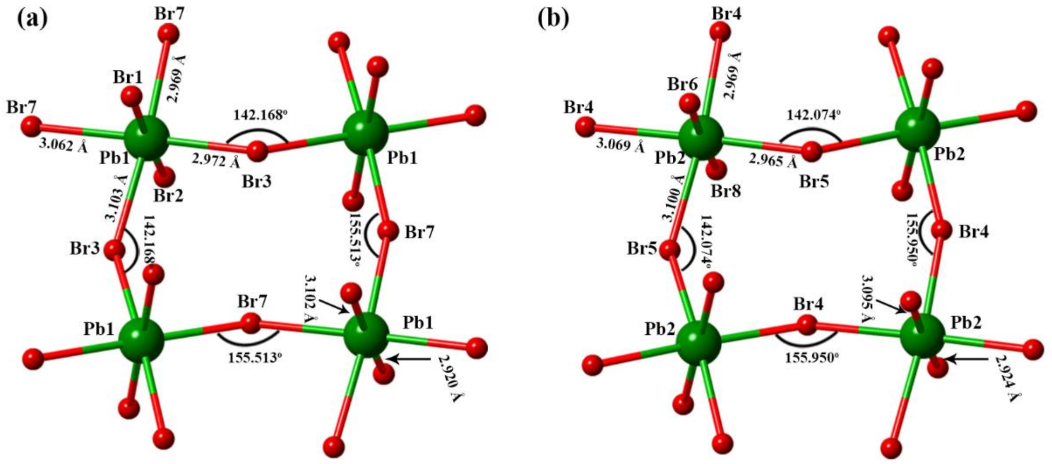

Figure S4. (a) and (b) Pb-Br bond lengths and Br-Pb-Br bond angles of **1-*R*** at LTP.

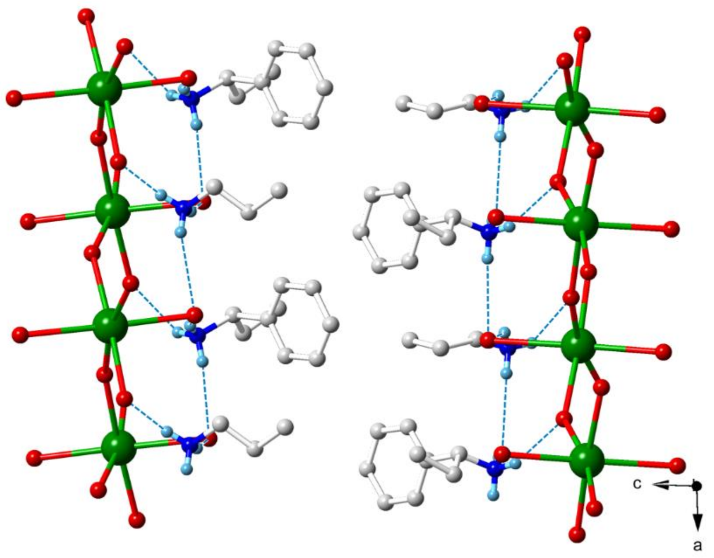

Figure S5. Hydrogen bonds between the organic cations and inorganic skeleton of **1-*R*** at LTP.

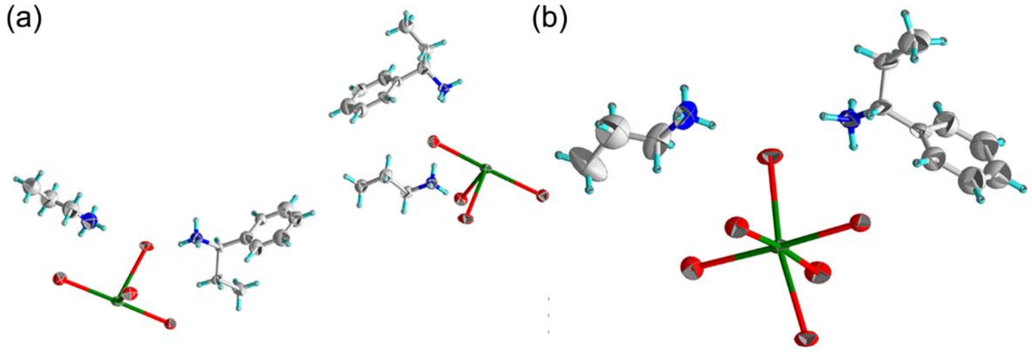

Figure S6. The thermal ellipsoid variation of **1-*R*** at LTP-300 K and HTP-380 K, respectively.

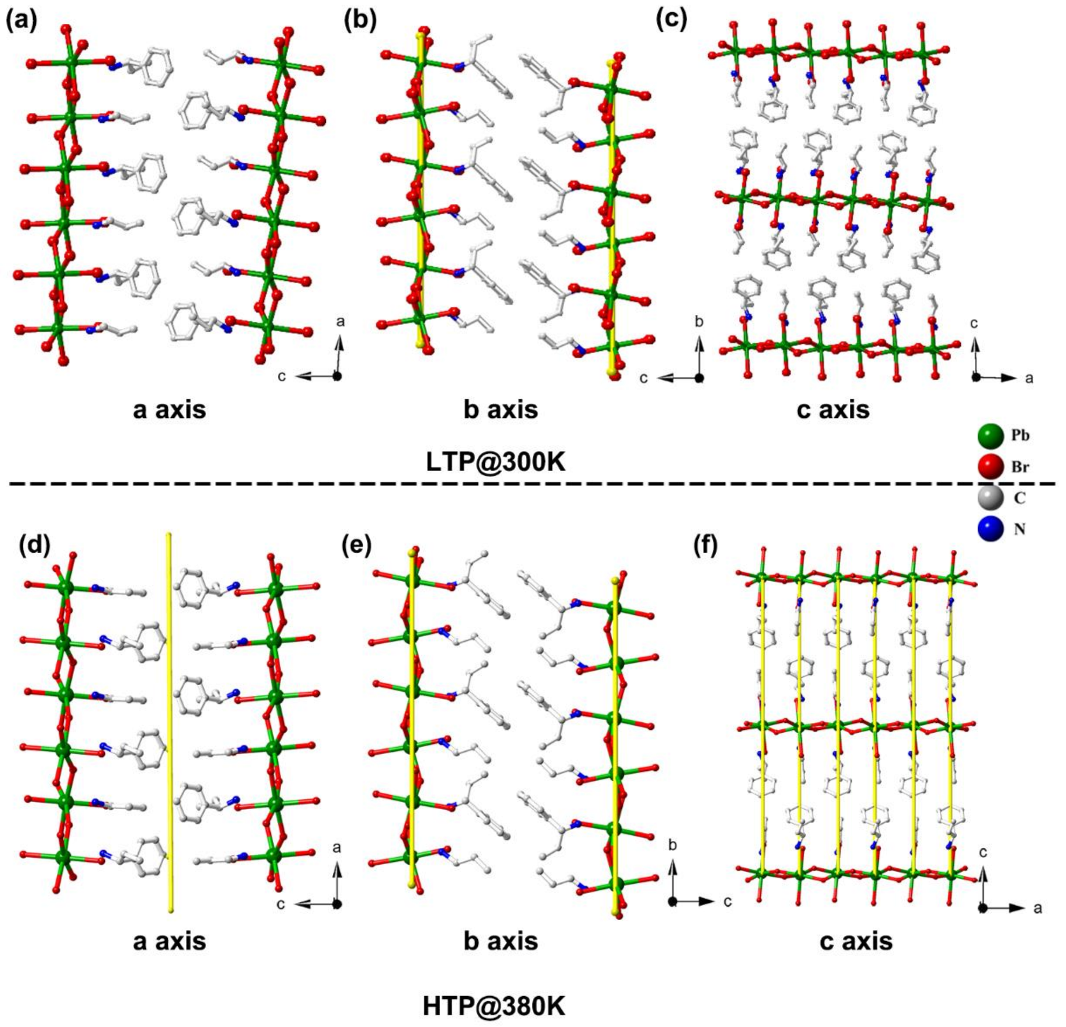

Figure S7. (a) and (c) the structure along *a*, *c*-axis in RT phase; (b) the 2_1 helical axis along *b*-axis in RT phase; (d)-(f) the 2_1 helical axis along a, b, c-axis in HTP phase, respectively. The yellow lines present the 2_1 helix axes.

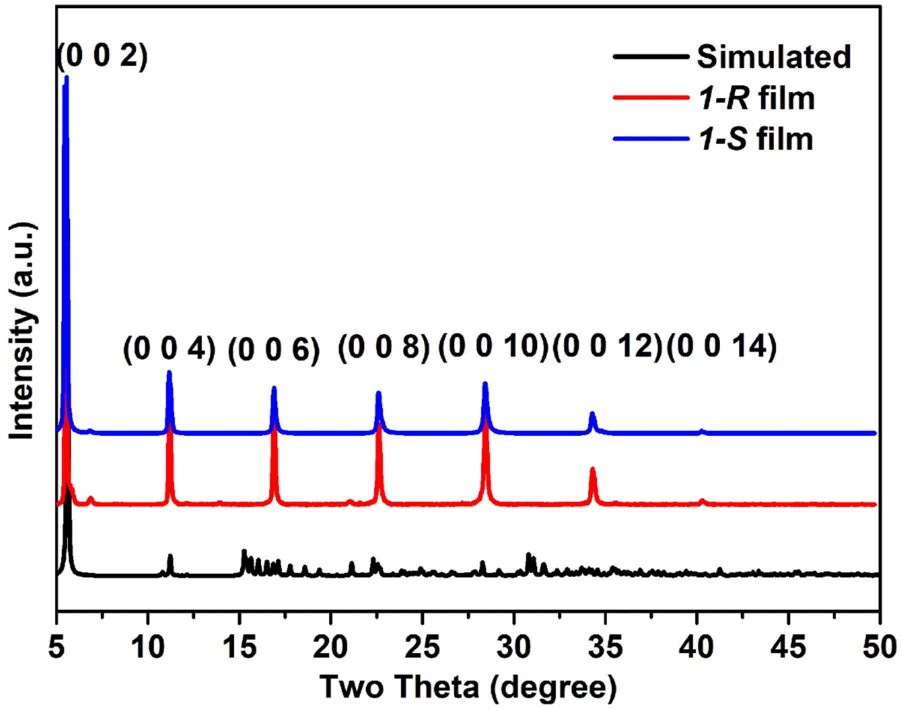

Figure S8. The PXRD of **1-*R*** and **1-*S*** thin films.

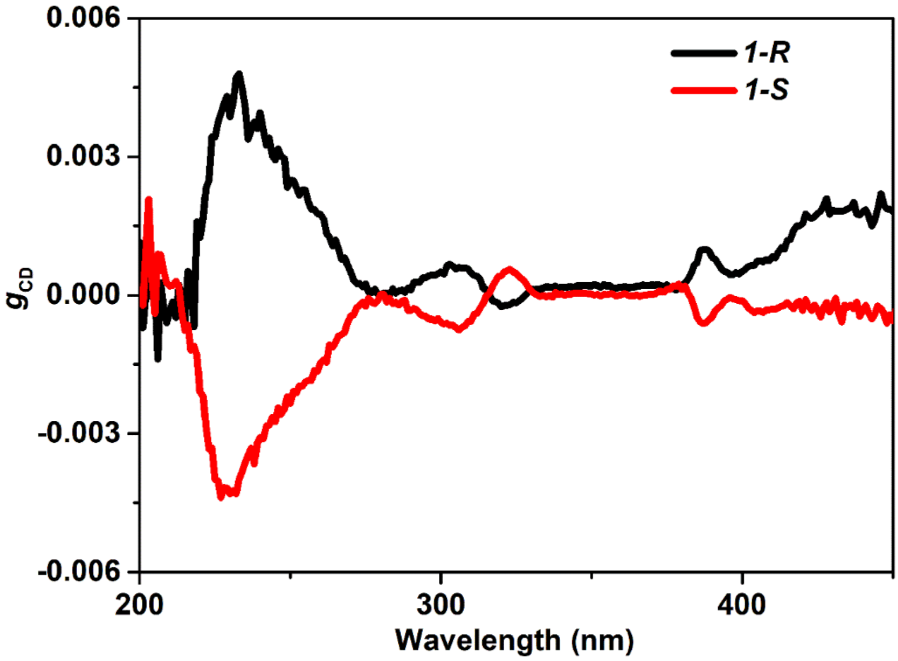

Figure S9. The g_{CD} spectra of **1-*R*/*S***.

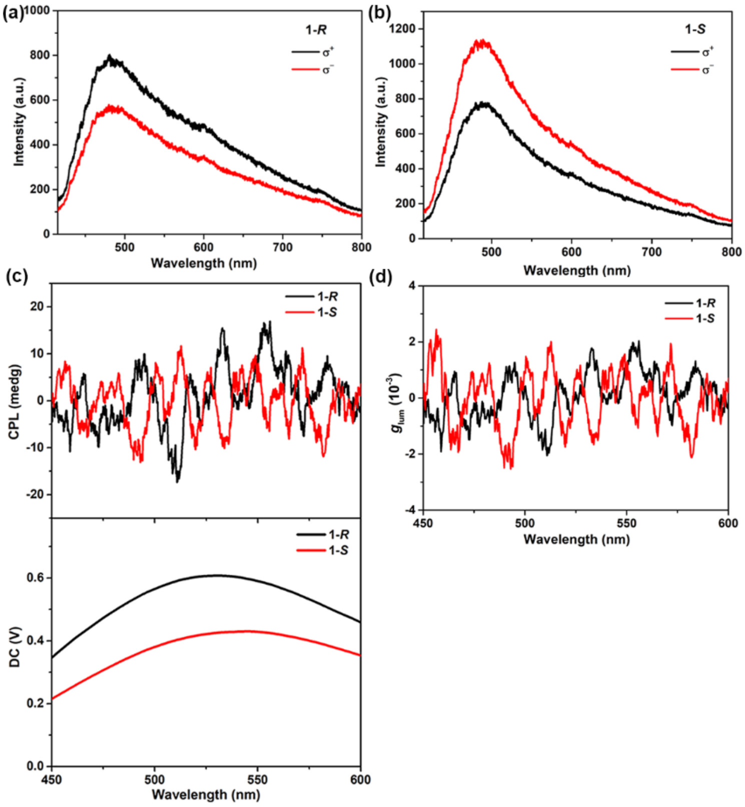

Figure S10. (a)-(b) circularly polarized PL emission of **1-*R*** and **1-*S*** crystals; (c) the CPL and DC spectra of **1-*R*** and **1-*S***; (d) The g_{lum} of **1-*R*** and **1-*S***.

To further investigate the selective left- and right-handed circularly polarized emission of **1-*R*/*S***, we simultaneously studied the CPL emission of **1-*R*** and **1-*S*** powder samples and bulk crystals under linearly polarized excitation light at 355 nm. (Figure S10c-d) As shown in Figure S10c-d, the **1-*R*** and **1-*S*** exhibit no distinguishable CPL emission, at least at room temperature.

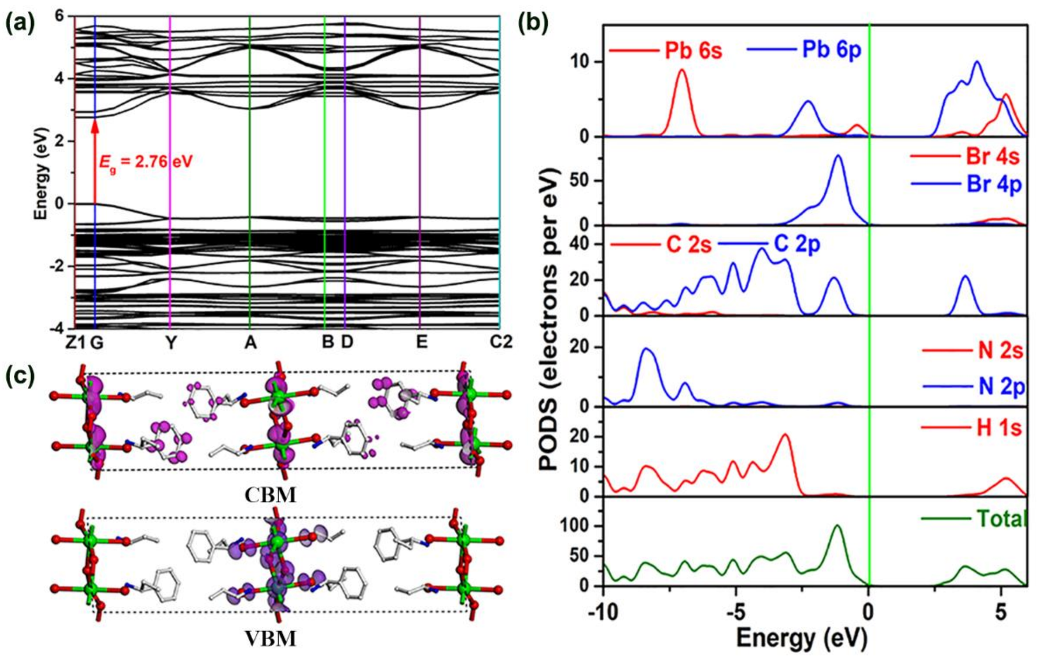

Figure S11. (a) The calculated band structure of **1-*R***; (b) The partial and total DOS profiles of **1-*R***; (c) The charge density isosurfaces for the VBM and CBM of **1-*R*** by using DFT method.

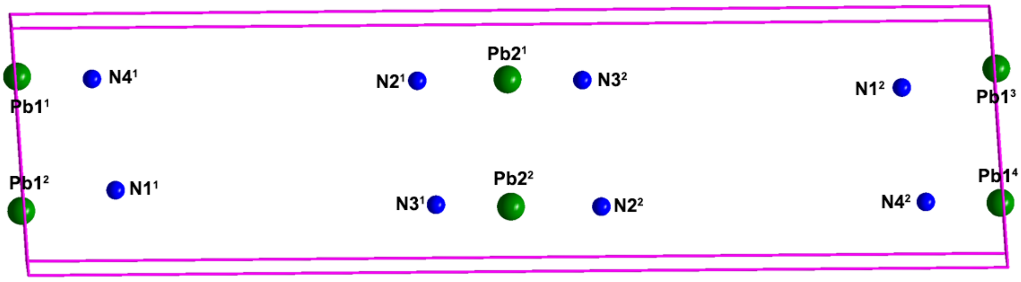

Figure S12. Distribution of Pb and Br atoms of **1-*R*** in a unit cell at LTP.

Based on the crystal structure data collected at 300 K, we select a unit cell and assume that the centers of the positive charges of the $(PPA\text{-}PA)^{2+}$ and the negative charges of the $(PbBr_4)^{2-}$ are located on the PPA, PA atoms and Pb atoms, respectively.

Table S1. **The atoms and atoms coordinate of Pb and N.**

Atoms	Atoms coordinate	
Pb1	$Pb1^1$ (0.24261, 0.16766, 0.99723)	$Pb1^2$ (0.75739, 0.66766, 1.00277)

	Pb1[3] (0.24261, 0.16766, -0.00277)	Pb1[4] (0.75739, 0.66766, 0.00277)
Pb2	Pb2[1] (0.25708, 0.40094, 0.49709)	Pb2[2] (0.74292, 0.90094, 0.50291)
N1	N1[1] (0.70898, 0.10901, 0.9046)	N1[2] (0.29102, 0.60901, 0.0954)
N2	N2[1] (0.22803, 0.95001, 0.5898)	N2[2] (77197, 0.45001, 0.4102)
N3	N3[1] (0.76398, 0.35901, 0.5788)	N3[2] (0.23602, 0.85901, 0.4212)
N4	N4[1] (0.22302, 0.71899, 0.9215)	N4[2] (0.77698, 0.21899, 0.0785)

The electric polarization (P_s) along *b*-axis can be estimated to be:

P_s={[-2e×(0.16766+0.66766+0.16766+0.66766)/2]+[-2e×(0.40094+0.90094)]+e×(0.10901+0.60901+0.95001+0.45001+0.35901+0.85901+0.71899+0.21899)} ×b/V

$=-3.6\times10^{-4}\times1.6\times10^{-19}\times8.472\times10^{-10}$ C m/(2129.5×10^{-30} m^3)

$=-2.291\times10^{-5}$ C m^{-2}

$|P_s|$= 2.291 nC cm^{-2}

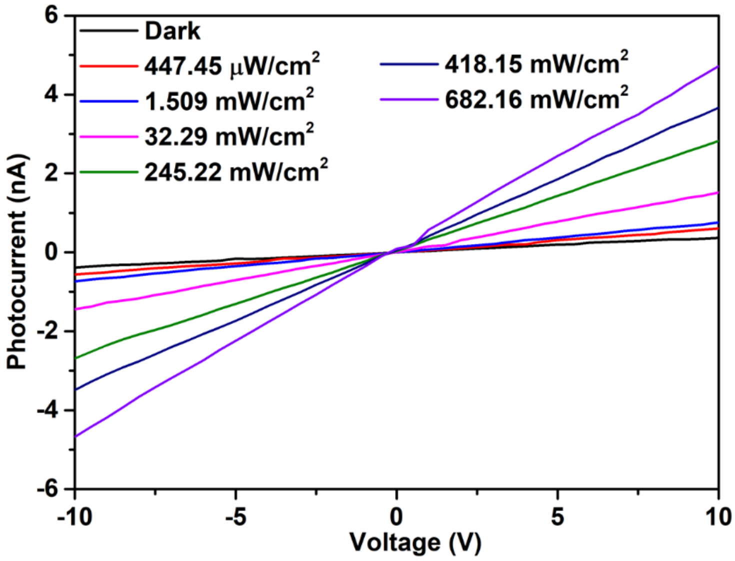

Figure S13. *I-V* curve of **1-*R*** SC device in the dark and 405 nm light illumination with varied intensities in HTP.

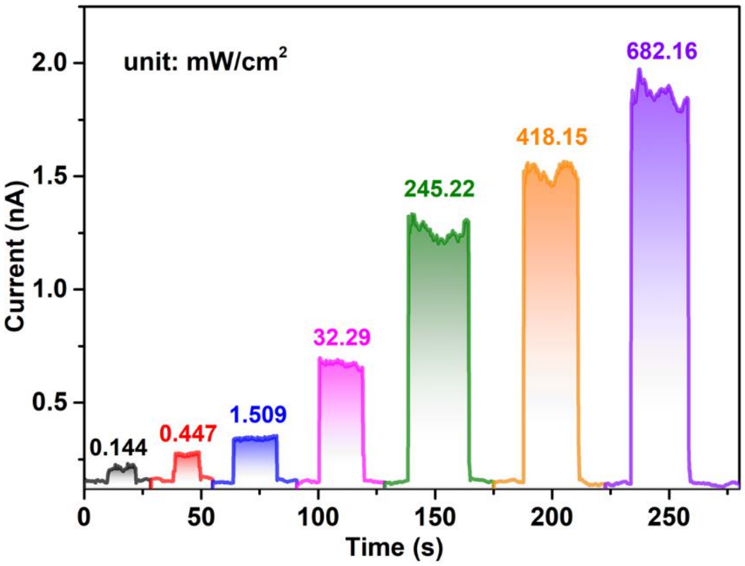

Figure S14. *I*-*t* curve of **1-*R*** SC device under light illumination with different intensities in HTP.

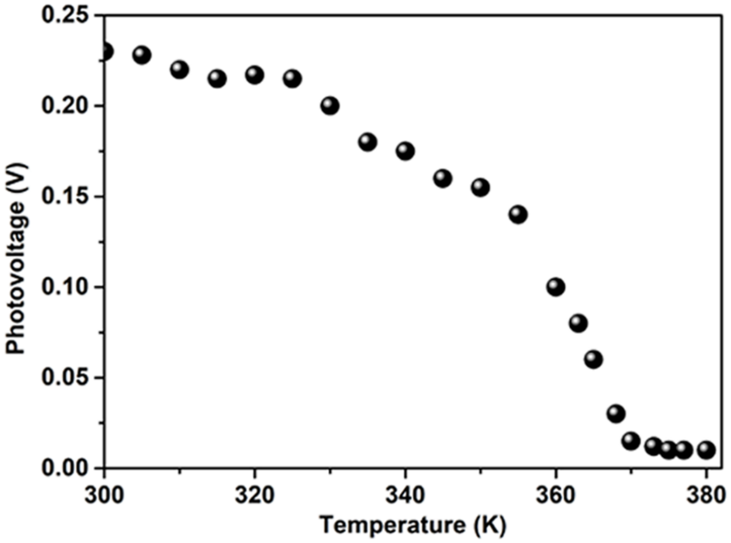

Figure S15. *V*-*T* curve under 682.16 mW/cm^2.

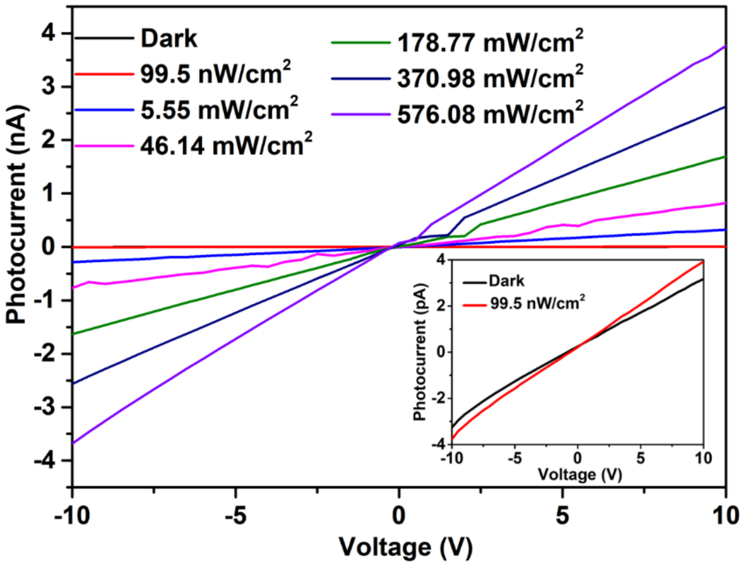

Figure S16. *I*-*V* curve of **1-*R*** SC device in the dark and 405 nm light illumination with varied intensities in LTP, the inner window is the *I*-*V* under weak light.

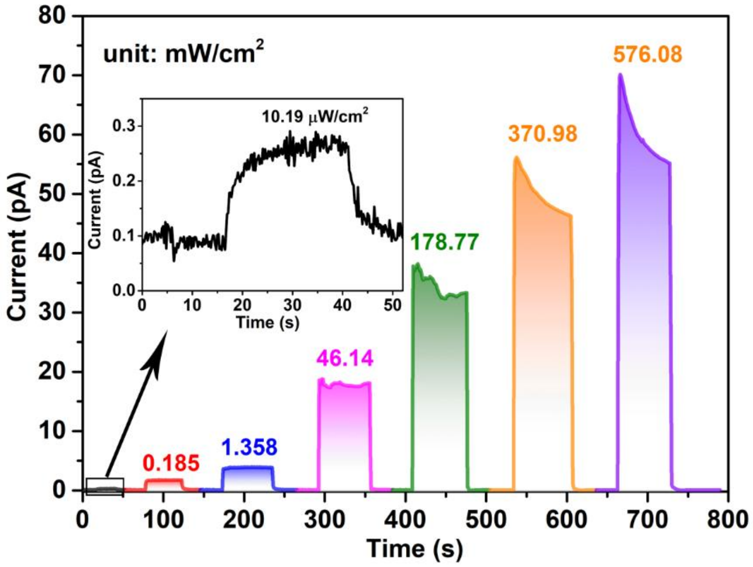

Figure S17. *I*-*t* curve of **1-*R*** SC device under light illumination with different intensities in LTP, the inner window is the *I*-*t* under weak light.

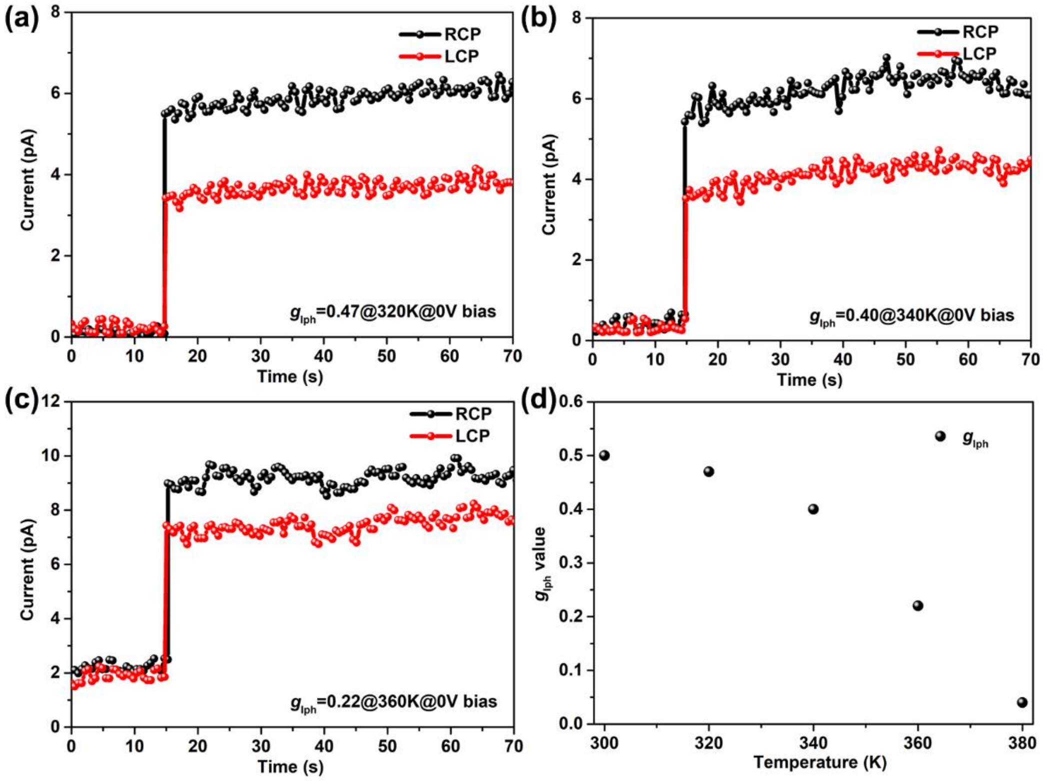

Figure S18. (a)-(c) the g_{Iph} value of CPL detection in 320K, 340K, and 360K, respectively; (d) the regulation of g_{Iph}.

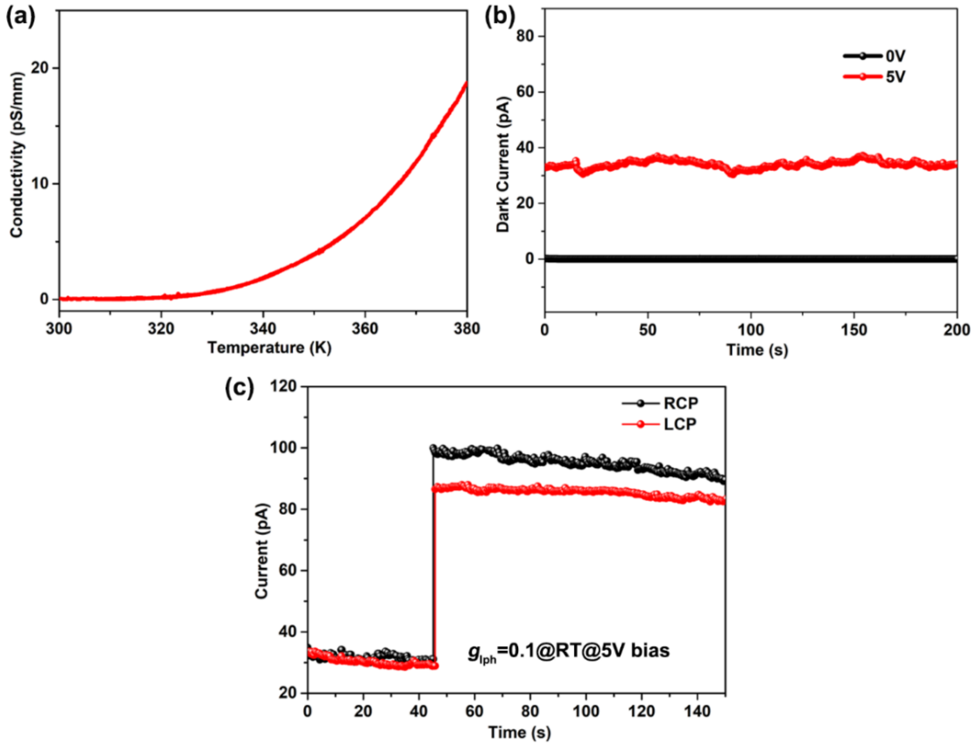

Figure S19. (a) the temperature-dependent conductivity of **1-*R***; (b) the dark current of **1-*R*** under 5V bias and 0V bias at room temperature; (c) CPL measurement at RT@5V-bias.

There are two main reasons why the current at 380K and 5V applied bias is larger than that at room temperature and 0V bias. 1. Under the effect of the built-in electric field generated by the polar photovoltaic effect (PPE), the dark current can be greatly reduced, and this can be proved by the **Figure S19b**. Specifically, at room temperature, the dark current at 5V bias is much higher than that at 0V bias. (**Figure S19b**) 2. Temperature-dependent conductivity tests have shown that the conductivity of **1-*R*** increases with temperature rise, (**Figure S19a**) which results in a decrease in resistance and a sharp increase in current value as the temperature increases. In addition, thermal effects have a significant impact on carrier transport, and more carrier density could be generated according to the Fermi-Dirac distribution at high temperatures, which may contribute to the generation of high current at high temperatures. (*ACS Nano* **2023**, *17*, 20502-20511; *Adv. Optical Mater.* **2022**, *10*, 2201378) Therefore, **1-*R*** device has a higher current value at 380K and 5V bias than that at RT and 0V bias.

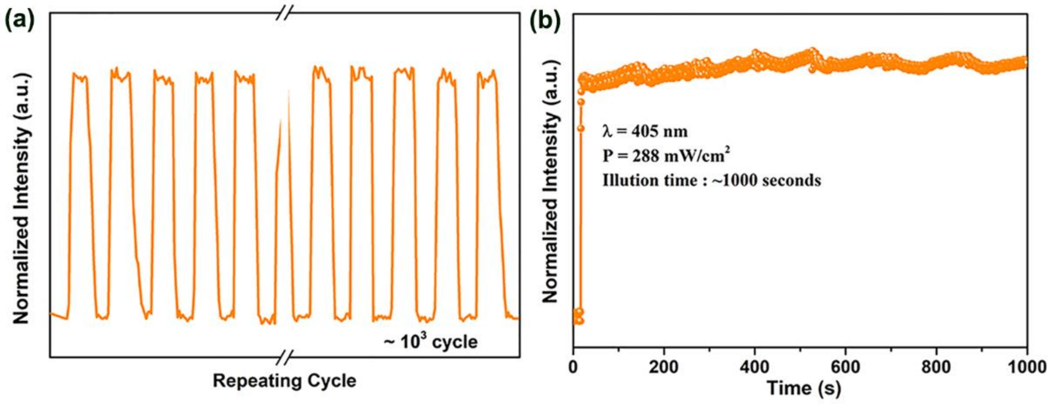

Figure S20. (a) the repeating cycles of **1-*R*** device at room temperature; (b) long-term photocurrent of **1-*R*** device under 5 V bias at 380 K.

Table S2. Crystal data for 1-*R* at LTP and HTP, and 1-*S*, respectively.

Compound	**1-*R* at LTP (300K)**	**1-*R* at HTP (380K)**	**1-*S***
Empirical formula	$H_{24}C_{12}N_2PbBr_4$	$H_{24}C_{12}N_2PbBr_4$	$H_{24}C_{12}N_2PbBr_4$
Formula weight	723.16	723.16	723.16
Temperature (*K*)	300 K	380 K	100 K
Space group	$P2_1$	$P2_12_12_1$	$P2_1$

Cell parameters	***a*** = 7.974(3) Å<br>***b*** = 8.472(3) Å<br>***c*** = 31.579(12) Å<br>***α*** = 90°<br>***β*** = 93.464(13)°<br>***γ*** = 90°	***a*** = 8.0130(4) Å<br>***b*** = 8.4981(4) Å<br>***c*** = 31.347(2) Å<br>***α*** = 90°<br>***β*** = 90°<br>***γ*** = 90°	***a*** = 7.8424(5) Å<br>***b*** = 8.3432(7) Å<br>***c***= 31.032(2) Å<br>***α*** = 90°<br>***β*** = 95.294(7)°<br>***γ*** = 90°
V (Å^3)	2129.5(13)	2134.6(2)	2021.8(3)
Z, $\rho_{cal.}$ (g/cm^3)	4, 2.256	4, 2.250	4, 2.376
F(000)	1328	1328	1328
Radiation λ (Mo/Ga Kα) (Å)	0.71073	0.71073	1.3405
Theta range (°)	2.559 to 24.998	2.483 to 24.996	2.530 to 60.187
Limiting indices	-9 ≤ h ≤ 9<br>-10 ≤ k ≤ 10<br>-37 ≤ l ≤ 37	-9 ≤ h ≤ 8<br>-10 ≤ k ≤ 10<br>-36 ≤ l ≤ 37	-10 ≤ h ≤ 10<br>-8 ≤ k ≤ 10<br>-39 ≤ l ≤ 40
Reflections collected /unique	27598 / 7384 [R(int) = 0.0961]	11262 / 3711 [R(int) = 0.0898]	13818 / 7711 [R(int)= 0.0511]
Data/restraints/parameter	7384 / 150 / 309	3711 / 8 / 165	7711 / 163 / 341
Final R indices [$I > 2\sigma(I)$][a]	R_1 = 0.0860, wR_2 = 0.2218	R_1 = 0.0771, wR_2 = 0.1539	R_1 = 0.0839,wR_2 = 0.2336
R indices (all data)	R_1 = 0.1124, wR_2 = 0.2380	R_1 = 0.1212, wR_2 = 0.1690	R_1 = 0.0889, wR_2 = 0.2416

[a]$R_1 = \Sigma||F_o| - |F_c||/\Sigma|F_o|$, $wR_2 = [\Sigma w(F_o^2 - F_c^2)^2/\Sigma(F_o^2)^2]^{1/2}$

Table S3. Bond lengths between metal halides for 1-*R* at LTP and HTP and 1-*S*, respectively.

For 1-*R* at LTP

Atom	Atom	Length/Å	Atom	Atom	Length/Å
Pb1	Br1	3.102(6)	Pb2	Br4#3	2.969(6)
Pb1	Br2	2.920(6)	Pb2	Br4	3.069(5)
Pb1	Br3#1	2.972(5)	Pb2	Br5#4	2.965(5)

Pb1	Br3	3.103(5)	Pb2	Br5	3.100(5)
Pb1	Br7	3.062(5)	Pb2	Br6	3.095(7)
Pb1	Br7#2	2.969(6)	Pb2	Br8	2.924(6)

For 1-*R* at HTP

Atom	Atom	Length/Å	Atom	Atom	Length/Å
Pb1	Br1#1	2.961(3)	Pb1	Br3#2	3.068(3)
Pb1	Br1	3.107(3)	Pb1	Br3	2.969(3)
Pb1	Br2	3.050(4)	Pb1	Br4	2.902(4)

For 1-*S*

Atom	Atom	Length/Å	Atom	Atom	Length/Å
Pb1	Br1	2.912(4)	Pb2	Br5	2.912(4)
Pb1	Br2#1	2.955(4)	Pb2	Br8	2.941(4)
Pb1	Br4	2.966(4)	Pb2	Br6#3	2.954(4)
Pb1	Br4#2	3.019(4)	Pb2	Br8#4	3.047(4)
Pb1	Br2	3.043(4)	Pb2	Br6	3.047(4)
Pb1	Br3	3.065(4)	Pb2	Br7	3.069(4)

Table S4. The figure-of-merits of some reported CPL detectors based on CHPs.

**Dimension**	**Materials**	**Anisotropy factor (g_{Iph})@wavelength**	**Power**	**Reference**
2D	(*R*-PPA)PAPbBr_4 single crystal at LTP	0.5@405nm	Self-powered@0 V	This work
2D	(R-MBA)$_2$Pb$_{0.9}$Sn$_{0.1}$$I_4$ film	0.44@500nm	Self-powered@0 V	1
2D	(R-PPA)EAPbBr_4 single crystal	0.42@266nm	Self-powered@0 V	2
2D	(*R*-PPA)EAPbCl_4 single crystal	0.4@266nm	Self-powered@0 V	3

2D	[(*R*)-β-MPA]$_4$AgBiI$_8$ single crystal	0.3@520nm	Self-powered@0 V	4
2D	(R-/S-PPA)EA$_2$Pb$_2$Br$_7$ single crystal	0.3@266nm	Self-powered@0 V	5
2D	(*S*-α-MBA)$_2$PbI$_4$ NW array	0.24@510nm	Bias-driven@5 V	6
2D	*S*-VPEA film	0.22@490nm	/	*7*
2D	(S-PPA)$_4$(IPA)$_6$Ag$_2$Bi$_4$I$_{24}$·2H$_2$O single crystal	0.21@520nm	Bias-driven@1 V	8
2D	[(*R*)-β-MPA]$_2$MAPb$_2$I$_7$ film	0.2@532nm	Bias-powered@5 V	9
2D	(*R*-*β*-MPA)EAPbBr$_4$ single crystal	0.19@405nm	Self-powered@0 V	10
2D	(NEA)$_2$(MA)$_{n-1}$Pb$_n$I$_{3n+1}$ film	0.15@405nm	Bias-powered@20 V	11
2D	(*R*-BPEA)$_2$PbI$_4$ single crystal	0.13@520nm	Bias-powered@-10 V	12
2D	(*R*/*S*-3AMP)PbBr$_4$	0.20@430 nm	Bias-powered@10 V	13
2D	(R-α-PEA)$_2$PbI$_4$ Nanowires	0.15@505nm LED	Bias-powered@5 V	14
2D	[(*R*)-β-MPA]$_2$PbCl$_4$ single crystal	0.1@266nm	Bias-powered@10 V	15
3D	3D Cs$_{0.05}$FA$_{0.5}$MA$_{0.45}$Pb$_{0.5}$Sn$_{0.5}$I$_3$ polycrystalline films incorporate chiral plasmonic gold nanoparticles (AuNPs)	0.55@808nm	self-powered@0V	16
3D	MAPbBr$_3$-*R*	0.39@405nm	Bias-powered@5 V	17
3D	(*R*/*S*-BPEA)EA$_6$Pb$_4$Cl$_{15}$ single crystal	0.28@320nm	Bias-powered@10 V	18
1D	(*R*/*S*-NEA)PbI$_3$ film	1.85@395nm	Bias-powered@-0.5 V	19

1D	(R-NEA)PbI_3 flake	0.294@405nm	Bias-powered@4 V	20
1D	(4-AMP)BiI_5 single crystal	0.24@405nm	Self-powered@0 V	21
1D	(*R*-$C_5H_{14}N$)PbI_3 microwire	0.23@405nm	Bias-powered@5 V	22
1D	[(*R*/*S*)-3-aminopiperidine]PbI_4 single crystal	0.21@1064nm	/	23
0D	(*R*/*S*-MBA)$_4Bi_2Br_{10}$ spiral microplates	0.58@1200nm	/	24
0D	(*R*-1-NEA)$_2CuCl_4$ film	0.41@880nm	/	25
2D/3D	[(*R*)-MPA]$_2MAPb_2I_7$/$MAPbI_3$ heterostructure single crystal	0.67@520nm	Self-powered@0 V	26
2D/3D	(*R*-BPEA)$_2PbI_4$/$MAPbI_3$ heterostructure	0.25@785nm	Self-powered@0 V	27
2D/3D	(R-α-PEA)$_2PbI_4$/$MAPbI_3$ heterostructure films	0.13@ 473 nm	Bias-powered@3 V	28
2D/2D	[(R)/(S)-β-MPA]$_2PbI_4$/[(R)/(S)-β-MPA]$_2MA_{n-1}Pb_nI_{3n+1}$ heterostructure NWs	0.38@510nm	Bias-powered@5 V	29
2D/Si	[(R)-MPA]$_2PbCl_4$/Si heterostructure	0.4@266nm	Self-powered@0 V	15
2D/Si	(R-MPA)$_2MAPb_2I_7$/Si heterostructure	0.34@520nm	Self-powered@0 V	30

References

[1] B. Yao, Q. Wei, Y. Yang, W. Zhou, X. Jiang, H. Wang, M. Ma, D. Yu, Y. Yang, Z. Ning, *Nano Lett.* **2023**, *23*, 1938-1945.
[2] T. Zhu, W. Weng, C. Ji, X. Zhang, H. Ye, Y. Yao, X. Li, J. Li, W. Lin, J. Luo, *J. Am. Chem. Soc.* **2022**, *144*, 18062-18068.

[3] T. Zhu, H. Wu, C. Ji, X. Zhang, Y. Peng, Y. Yao, H. Ye, W. Weng, W. Lin, J. Luo, *Adv. Optical Mater.* **2022**, 2200146.
[4] D. Li, X. Liu, W. Wu, Y. Peng, S. Zhao, L. Li, M. Hong, J. Luo, *Angew. Chem. Int. Ed.* **2021**, *60,* 8415-8418.
[5] T. Zhu, K. Zhang, C. Ji, X. Zhang, H. Ye, Y. Zou, J. Luo, *Small* **2022**, 2203571.
[6] Z. Liu, C. Zhang, X. Liu, A. Ren, Z. Zhou, C. Qiao, Y. Guan, Y. Fan, F. Hu, Y. S. Zhao, *Adv. Sci.* **2021**, 2102065.
[7] Y. Zhao, X. Yin, Z. Gu, M. Yuan, J. Ma, T. Li, L. Jiang, Y. Wu, Y. Song, *Adv. Funct. Mater*. **2023**, 2306199.
[8] Z.-K. Zhu, T. Zhu, J. Wu, S. You, P. Yu, X. Liu, L. Li, C. Ji, J. Luo, *Adv. Funct. Mater.* **2023**, 2214660.
[9] L. Wang, Y. Xue, M. Cui, Y. Huang, H. Xu, C. Qin, J. Yang, H. Dai, M. Yuan, *Angew. Chem. Int. Ed.* **2020**, *59*, 6442-6450.
[10] W. Wu, L. Li, D. Li, Y. Yao, Z. Xu, X. Liu, M. Hong, J. Luo, *Adv. Optical Mater.* **2022**, 2102678.
[11] T. Liu, W. Shi, W. Tang, Z. Liu, B. C. Schroeder, O. Fenwick, M. J. Fuchter, *ACS Nano* **2022**, *16*, 2682-2689.
[12] Y. Peng, X. Liu, L. Li, Y. Yao, H. Ye, X. Shang, X. Chen, J. Luo, *J. Am. Chem. Soc.* **2021**, *143,* 14077-14082.
[13] C.-C. Fan, X.-B. Han, B.-D. Liang, C. Shi, L.-P. Miao, C.-Y. Chai, C.-D. Liu, Q. Ye, W. Zhang, *Adv. Mater.* **2022**, *34*, 2204119.
[14] Y. Zhao, Y. Qiu, J. Feng, J. Zhao, G. Chen, H. Gao, Y. Zhao, L. Jiang, Y. Wu, *J. Am. Chem. Soc.* **2021**, *143*, 8437-8445.
[15] X. Zhang, W. Weng, L. Li, H. Wu, Y. Yao, Z. Wang, X. Liu, W. Lin, J. Luo, *Small* **2021**, *17,* 2102884.
[16] H. Kim, R. M. Kim, S. D. Namgung, N. H. Cho, J. B. Son, K. Bang, M. Choi, S. K. Kim, K. T. Nam, J. W. Lee, J. H. Oh, *Adv. Sci.* **2022**, *9*, 2104598.
[17] G. Chen, X. Liu, J. An, S. Wang, X. Zhao, Z. Gu, C. Yuan, X. Xu, J. Bao, H.-S. Hu, J. Li, X. Wang, *Nat. Chem.* **2023**, DOI: 10.1038/s41557-023-01290-2.
[18] Q. Guan, T. Zhu, Z.-K. Zhu, H. Ye, S. You, P. Xu, J. Wu, X. Niu, C. Zhang, X. Liu, J. Luo, *Angew. Chem. Int. Ed.* **2023**, *62*, e202307034.
[19] A. Ishii, T. Miyasaka, *Sci. Adv.* **2020**; *6* : eabd3274.
[20] M. Li, F. Fang, X. Huang, G. Liu, Z. Lai, Z. Chen, J. Hong, Y. Chen, R.-J. Wei, G.-H. Ning, K. Leng, Y. Shi, B. Tian, *Chem. Mater.* **2022**, *34*, 2955-2962.

[21] T. Zhu, J. Bie, C. Ji, X. Zhang, L. Li, X. Liu, H.-Y. Huang, W. Fa, S. Chen, J. Luo, *Nat. Commun.* **2022**, 13, 7702.

[22] Y. Zhao, X. Li, J. Feng, J. Zhao, Y. Guo, M. Yuan, G. Chen, H. Gao, L. Jiang, Y. Wu, *Giant*, **2022**, *9*, 100086.

[23] D. Fu, J. Xin, Y. He, S. Wu, X. Zhang, X.-M. Zhang, J. Luo, *Angew. Chem. Int. Ed.* **2021**, *60*, 20021-20026.

[24] X. Fu, Z. Zeng, S. Jiao, X. Wang, J. Wang, Y. Jiang, W. Zheng, D. Zhang, Z. Tian, Q. Li, A. Pan, *Nano Lett.* **2023**, *23*, 606-613.

[25] Z. Guo, J. Li, J. Liang, C. Wang, X. Zhu, T. He, *Nano Lett.* **2022**, *22*, 846-852.

[26] X. Zhang, X. Liu, L. Li, C. Ji, Y. Yao, J. Luo, *ACS Cent. Sci.* **2021**, *7*, 1261-1268.

[27] X. Zhang, H. Ye, L. Liang, X. Niu, J. Wu, J. Luo, *ACS Appl. Mater. Interfaces* **2022**, *14*, 36781-36788.

[28] L. Tao, W. Tang, M. Yan, L. Ding, J. Wei, L. Wang, L. Li, L. Li, D. Yang, Y. Fang, *J. Mater. Chem. C*, **2023**, *11*, 12392-12399.

[29] Y. Zhao, Z. Zhou, X. Liu, A. Ren, S. Ji, Y. Guan, Z. Liu, H. Liu, P. Li, F. Hu, Y. S. Zhao, *Adv. Optical Mater.* **2023**, 2301239.

[30] J. Wu, X. Zhang, S. You, Z.-K. Zhu, T. Zhu, Z. Wang, R. Li, Q. Guan, L. Liang, X. Niu, J. Luo, *Small*, **2023**, *19*, 2302443.
